# Measurement properties of the Activities-specific Balance Confidence (ABC) scale in community-dwelling adults aged 50 years and older: a COSMIN systematic review

**DOI:** 10.1186/s41687-026-01119-9

**Published:** 2026-06-19

**Authors:** Hadi Kooshiar, Dan Song, Elissa Dabkowski, Karen Missen

**Affiliations:** 1 Department of Nursing Practice, College of Nursing and Health Sciences, Texas A&M University–Corpus Christi, Corpus Christi, TX USA; 2 Institute of Health and Wellbeing, Federation University, Churchill, Australia

**Keywords:** Accidental falls, Activities-specific balance confidence, Balance confidence, COSMIN systematic review

## Abstract

**Aim:**

To systematically review and grade the measurement properties of the Activities-specific Balance Confidence (ABC) scale in community-dwelling older adults using the COSMIN framework.

**Methods:**

Five electronic databases were searched through July 2025 from inception. Eligible studies included adults aged 50 years or older and examined the ABC scale in any version or translation. Methodological quality was evaluated using the COSMIN Risk of Bias checklist, and the certainty of the evidence was graded with the COSMIN-adapted GRADE approach. The protocol was registered with PROSPERO (ID: CRD420251178952).

**Results:**

A total of 22 studies were included, covering the ABC-16 (*n* = 16), ABC-6 (*n* = 5), and ABC-S (*n* = 1) in 11 languages. Internal consistency (α = 0.86–0.99) and test–retest reliability (ICC = 0.82–0.99) were consistently sufficient with moderate certainty. Structural and construct validity were generally supported. In contrast, content validity was primarily assessed during cross-cultural adaptations, measurement error was seldom assessed, and cross-cultural validity and responsiveness were rarely tested. The Arabic ABC-16 showed the strongest overall evidence and was categorized as ready for use (Category A). Versions in Chinese-Canadian, Cantonese, Mandarin ABC-6, Brazilian Portuguese, Persian, German, Urdu, Sepedi, Chinese, English (Canadian), and Hindi demonstrated promising properties but require local validation (Category B). Dutch and Mandarin ABC-16 versions as well as the earlier Portuguese version did not meet current COSMIN standards and require re-validation (Category C).

**Conclusion:**

The ABC scale generally demonstrates strong reliability and validity of balance confidence in older adults. The ABC-16 remains the most comprehensive version, while the ABC-6 provides an efficient screening tool. Category A versions can be considered for use in clinical and research settings. Future work should focus on responsiveness, cross-cultural invariance, and interpretability to strengthen clinical utility.

## Introduction

Falls are a major cause of injury, disability, loss of independence, and healthcare expenses among older adults. Annually, one in four adults over the age of 65 experiences falls, leading to fractures, decreased mobility, and an estimated $50 billion in treatment costs in the U.S. alone [1, 2]. In 2019, falls accounted for more than 80% of hip fracture–related deaths and nearly 90% of emergency visits and hospitalizations related to such injuries [3]. Despite the magnitude of this problem, many older adults delay participation in fall prevention until after experiencing an injury, underscoring the urgent need for proactive, community-based screening to identify at-risk individuals before falls happen [4].

Although various physiological assessment tools, such as the Johns Hopkins Fall Risk Assessment Tool [FRAT] [5] and the Peninsula Health FRAT [6], exist for falls screening, they primarily focus on physical risk factors. In contrast, psychosocial and behavioral factors, such as fear of falling, balance confidence, and self-efficacy, are equally important contributors to fall risk but are often overlooked [7]. Patient-reported outcome measures (PROMs) are particularly effective in capturing these psychological and behavioral dimensions, offering valuable insights into fall risk factors [8, 9].

The ABC scale, created by Powell and Myers in 1995, is among the most commonly used PROMs for assessing balance confidence in older adults. The original 16-item form (ABC-16) requires respondents to rate their confidence in performing daily activities, scored 0–100 [10]. Shorter versions like the ABC-6 and ABC-Short versions have been developed later for use in clinical and research contexts [11].

Although the ABC scale is widely used in research and clinical practice, evidence regarding its measurement properties across various languages and cultures remains inconsistent. Only one previous systematic review, which included just three studies [12], has evaluated the ABC. Since then, comprehensive synthesis has yet to be conducted. The COnsensus-based Standards for the selection of Health Measurement Instruments (COSMIN) framework now offers internationally accepted standards for assessing PROMs, including reliability, validity, content validity, cross-cultural adaptation, and interpretability.

Prior COSMIN-based work has already appraised falls-efficacy–related PROMs in community-dwelling older adults. In particular, Soh et al. [13] conducted a COSMIN systematic review across 18 instruments, including the ABC-15, and evaluated structural validity sufficient for several widely-used instruments while noting limited and low-quality evidence for content validity and a need for stronger cross-cultural development. To the best of our knowledge, no single review has provided a dedicated, single-instrument COSMIN synthesis of the ABC (all versions/languages) across the full set of measurement properties with practice-ready recommendations. Accordingly, this review offers a comprehensive COSMIN appraisal of the ABC’s psychometric properties in community-dwelling adults. Given the widespread use of the ABC scale, this review is necessary to provide practice-ready, COSMIN-based recommendations across all versions and cultural adaptations.

## Methods

This review adhered to the COSMIN methodology for systematic reviews of PROMs [14], 2024). No meta-analysis was done due to the heterogeneity of the data. The protocol was registered prospectively in the PROSPERO International Register of Systematic Reviews (registration number: CRD420251178952). The COSMIN framework provides clear guidelines for evaluating PROMs, focusing on four main areas: the construct being assessed (ABC scale), the target population (Community older adults), the type of instrument (self-reported), and its measurement properties. Systematic reviews following this framework typically prioritize the assessment of key psychometric properties such as content validity, structural validity, internal consistency, reliability, and responsiveness [15]. Using COSMIN’s eight-step process, the review began by defining the research question and establishing the inclusion criteria. A detailed search strategy was then implemented across major databases to find relevant studies. Retrieved citations were screened based on predefined inclusion and exclusion criteria, and full texts were evaluated for eligibility. Data extracted included study design, participant details, instrument versions, cultural adaptations, and psychometric outcomes. Each study was evaluated for methodological quality using the COSMIN Risk of Bias checklist [14], which employs the “worst score counts” approach to prevent strong design features from masking weaknesses.

Measurement properties were evaluated according to the COSMIN criteria for good measurement properties. For example, internal consistency was considered sufficient if Cronbach’s α ≥ 0.70; reliability if ICC ≥ 0.70; and structural validity if confirmatory factor analysis fit indices met recommended thresholds. Results were classified as sufficient (+), insufficient (–), inconsistent (±), or indeterminate (?). Interpretability indices (e.g., standard error of measurement, minimal detectable change, cutoffs, floor/ceiling effects) and feasibility (e.g., administration time, burden) were extracted but not rated for quality.

The certainty of the evidence for each measurement property was graded using the COSMIN-adapted Grading of Recommendations Assessment, Development and Evaluation (GRADE) approach. Downgrades were applied for risk of bias, inconsistency, imprecision, or indirectness, and final certainty ratings were classified as high, moderate, low, or very low. Recommendations were formulated for each version of the ABC scale based on the balance of evidence across measurement properties [16]. Instruments were then classified into three recommendation levels: Category A (ready for use), Category B (promising but requiring further validation), and Category C (not recommended until re-validated).

### Eligibility studies

Studies qualified for inclusion if they assessed the (ABC) scale or its variants (e.g., ABC-16, ABC-6). Eligible research focused on community-dwelling adults aged 50 or older and reported outcomes related to falls, balance confidence, or fear of falling. Although ≥ 60 years is commonly used, a ≥ 50-year threshold was applied to align with prior falls research and to ensure inclusion of relevant validation studies. To be included, articles needed to be full-text, peer-reviewed publications that reported on at least one psychometric property of the ABC, such as validity or reliability. Translational studies examining fall risk perception in community settings were also considered eligible. Studies were excluded if they involved older adults living in nursing homes or assisted living facilities, or if the study populations were primarily defined by neurological or severe balance-impairing conditions (e.g., Parkinson’s disease, stroke, peripheral vestibular dysfunction, or lower-limb amputation). Common chronic conditions were not used as exclusion criteria unless they defined the study population. Additionally, discussion papers and intervention studies in which the ABC scale was used solely as an outcome measure, without reporting measurement properties, were excluded.

### Develop the literature search

A comprehensive search strategy was developed to identify relevant studies. Five electronic databases were searched from inception through July 2025: CINAHL, ProQuest, PubMed, PsycINFO, and EMBASE. Consistent with Cochrane methodology and consensus recommendations, PubMed and EMBASE were considered the minimum core databases, and Google Scholar was searched as a supplementary source to maximize coverage and was included in the identification stage; however, it did not yield additional eligible studies. No date restrictions were applied, in line with COSMIN guidance for PROM reviews.

The search strategy combined controlled vocabulary and keywords related to the ABC scale and falls in older adults. Search terms included “ABC scale,” “Activities-specific Balance Confidence scale,” “falls screening,” “balance confidence,” “accidental falls,” “older adults,” “elderly,” “community dwelling,” “psychometrics,” and “fall risk.” Boolean operators and database-specific syntax were applied to ensure sensitivity and precision.

Two reviewers, HK and DS, independently searched and screened all titles and abstracts. Duplicates were removed before screening. Any disagreements were resolved through discussion and, when necessary, by a third reviewer. Full-text articles were then retrieved and assessed for eligibility against the inclusion and exclusion criteria. Only studies meeting all criteria were included in the final review. The initial search identified 325 records, of which 29 were retained for full-text review and 22 were ultimately included in the synthesis.

### Perform the search (study selection and screening)

In line with Cochrane methodology, the term risk of bias refers to how reliable study outcomes are, considering their design and implementation [17]. To assess this, the methodological quality of each included study was evaluated using the COSMIN Risk of Bias checklist [14]. This tool is specifically created for PROMs and incorporates standards related to study design and statistical methods used to assess measurement properties.

The checklist evaluates key psychometric domains and rates each as very good, adequate, doubtful, or inadequate. For each measurement property, an overall judgment was derived using the COSMIN “worst score counts” principle, which prioritizes the lowest rating to ensure that methodological weaknesses are not overlooked. Results were then categorized as sufficient (+), insufficient (−), or indeterminate (?).

Because the focus of this review was exclusively on the (ABC) scale, all measurement properties were appraised according to COSMIN standards.

## Results

### Study selection and characteristics

**Fig. 1:**
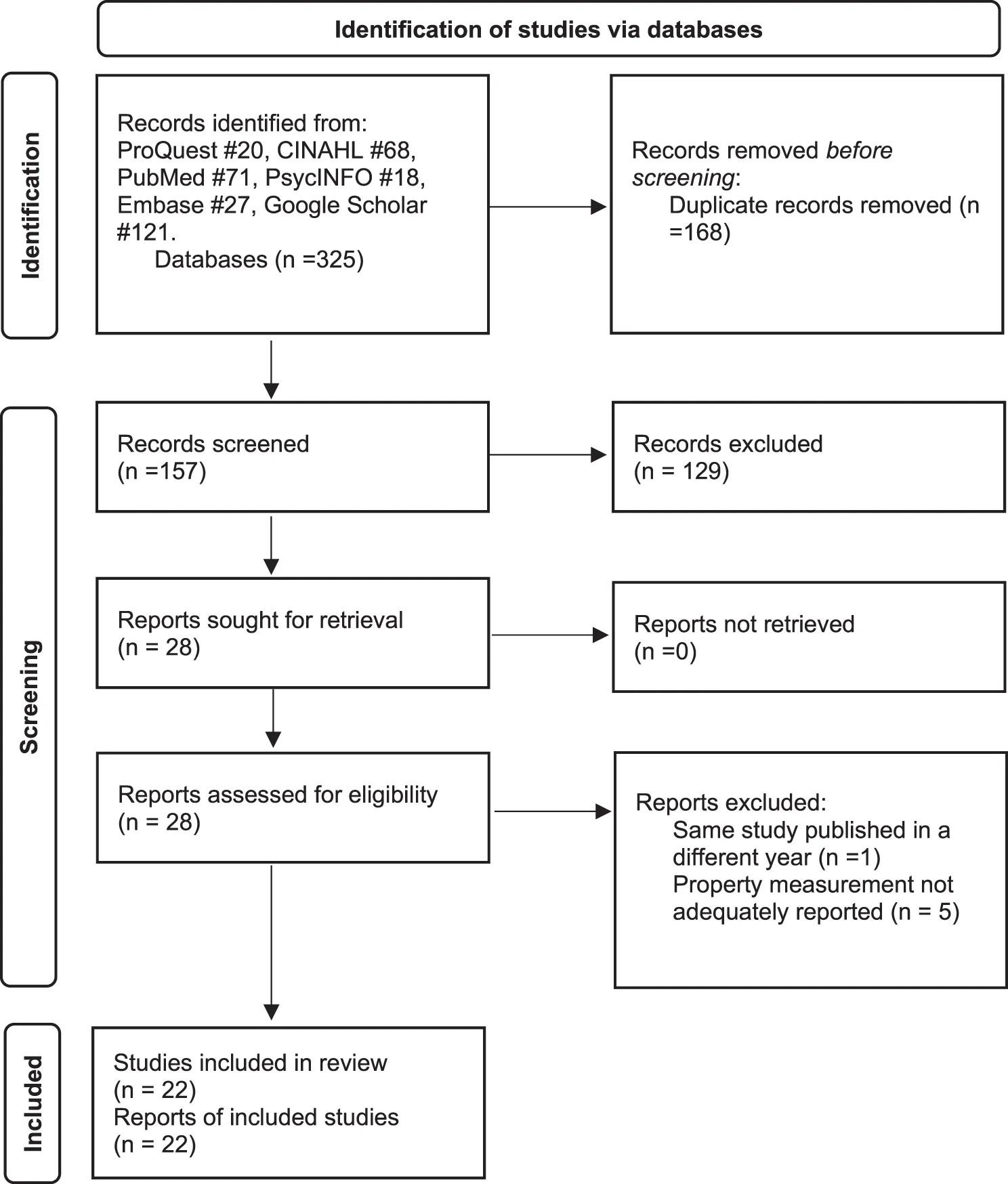
PRISMA 2020 flow diagram

The database search yielded 325 records, of which 22 studies met the inclusion criteria (Fig. 1, PRISMA flowchart). These included 16 studies of the ABC-16, five of the ABC-6, and one of the ABC-S. Versions were available in 11 languages across five continents, including Arabic, Urdu, Persian, Mandarin, Cantonese, Korean, German, Brazilian Portuguese, Sepedi, Hindi, and English/Canadian (Table 1). Sample sizes ranged from 35 to 384 participants, and most studies included older adults with mean ages generally above 60–65 years. All studies were conducted in community-dwelling older adults.

**Table 1:** Study characteristics of included studies

Authors (Year)	Country/Language	ABC Version	N	Aim	Inclusion/Exclusion	Comparators	Properties	Key Statistics	Results
[18]	Korea (Korean)	ABC-16	90	Validate Korean FES & ABC in community older adults	≥60y; community-dwelling; fall in past 3y/exclude missing items	FES, SF‑8, Katz ADL	IC; Rel; HT	α = 0.9623; test–retest *r* ≈ 0.824	Reliable & valid for Korean older adults; strong correlations with FES and QoL
[19]	Netherlands (Dutch)	ABC‑NL (16 + 7 dual-task)	246	Psychometrics of Dutch ABC; add complex/dual task items	GISSA participants; relatively healthy/MMSE < 17 excluded	Falls, activity avoidance, PASE/PSE, balance tests	SV; IC; HT	α = 0.88–0.95 (23/7/16 items)	Satisfactory structure & internal consistency; dual-task items feasible
[20]	Canada (Chinese‑Canadian)	ABC‑16 (Chinese & English)	71	Translate the ABC scale to Chinese; assess reliability across versions	Chinese immigrants > 5y in Canada, >50y/neurological dx or incomplete data	Demographics; falls; medications; assistive devices; Changes in health status	SV; IC; Rel; HT; CCV	α = 0.98; ICC total = 0.87–0.88	Very strong reliability; cross-language consistency; content validity under-described
[21]	Hong Kong (Cantonese)	ABC‑16 (ABC‑C)	100	Translate & examine psychometrics of ABC‑C	≥65y; independent indoor ambulation/communication deficits; MMSE < 18	—	CV; SV; IC; Rel; HT	α = 0.97; ICC = 0.99; factor structure satisfactory	Excellent reliability & structure; culturally relevant
[22]	Canada (English/French)	ABC‑S (simplified; ~15 items, IRT)	200	Develop simplified ABC; test internal consistency & convergent validity	≥60y; community-dwelling; able to join exercise program/cognitive deficits	Balance tests; fear of falling; falls history	CV; SV; IC; HT	Reliability index≈0.86; good convergent validity	Valid & reliable simplified version; needs replication
[23]	USA (English)	ABC‑16 (vestibular risk cohort)	263	Compare ABC vs SAFE: reliability, validity, responsiveness	Community-dwelling; postural instability + ≥1 risk factor/terminal illness; heavy exercisers	SAFE; Worry; GDS; SF-36; BBT; TUG; gait speed; falls	SV; IC; Rel; HT; RESP	α = 0.95 (baseline); ABC–SAFE correlated	Strong internal consistency & validity; some responsiveness evidence
[24]	USA (English)	ABC‑6 (short) vs ABC‑16	35	Validate ABC‑6; compare to ABC‑16; relate to falls	≥60y; ambulatory; stand 30s/neuro dz; unstable medical; pain; cognitive impairment	UST; FR; TUG; MSL; 12‑mo falls history	SV; IC; Rel; HT; RESP(+L)	ICC: 0.76 (ABC‑16), 0.82 (ABC‑6)	ABC‑6 valid/reliable; stronger associations with falls than ABC‑16
[25]	China (Mandarin)	ABC‑16	61	Adapt Mandarin ABC; test reliability & validity	Able to stand ≥ 30s/vestibular, cardiopulmonary, severe bone dz, etc.	Original ABC‑16	IC; Rel; HT	α = 0.94; ICC test–retest = 0.98; inter‑rater = 0.96	Reliable & valid; supports use in mainland China
[26]	Brazil (Brazilian‑Portuguese)	ABC‑16	40	Translation, cross‑cultural adaptation, intra/inter‑rater reliability	Healthy; ≥60y; speak/read Portuguese; ≥4y education	—	Rel; CCV	ICC total≈0.80	Good reliability for Brazilian version
[27]	Germany (German)	ABC‑D6 and ABC‑D16	384	Compare reliability/validity of ABC‑D6 vs ABC‑D16	≥50y; ambulatory/cardio‑pulm terminal/infectious; neuro dz; cognitive impairment	TUG; one‑leg stance; 10‑m walk; FAB; PASE; SF‑36; fitness; TMT; falls	SV; IC; HT; Rel	α 0.92–0.95 (D16), 0.83–0.91 (D6)	Excellent reliability/validity; ABC‑6 efficient
[28]	USA (English)	ABC‑16	44	Evaluate test–retest reliability & internal consistency	≥65y; ambulatory within home/required physical assistance to walk excluded	—	IC; Rel	ICC = 0.879; α = 0.973	Reliable & internally consistent
[29]	Iran (Persian)	ABC‑16 (ABC‑P)	142	Translation, cultural adaptation, reliability & validity	≥60y; Persian speaking; no cognitive problems; independent ADLs	—	CV; IC; Rel; CCV; HT	α = 0.96; ICC = 0.97	High validity & reliability; culturally appropriate
[30]	USA (English)	ABC‑6 (underserved)	251	Examine validity & reliability of ABC‑6 in underserved adults	≥50y; ambulatory; able to follow instructions	CFRSI; ETGUG; FR; PASE; medication/vision/environment subscales; falls	IC; Rel; HT	Supports discrimination fallers vs non‑fallers	Useful brief measure; predictive of falls risk
[31]	India (Hindi)	ABC‑H (ABC‑16 Hindi)	100	Translate/adapt Hindi ABC; assess psychometrics; normative values	60–86y; read/speak Hindi/exclude recent PT; cognitive/psychotic problems	FES‑I (Hindi)	CV; IC; Rel; HT	α = 0.99; item ICC = 0.78–0.95; r(ABC‑H,FES‑I)=−0.62 (neg.)	Acceptable properties; suitable for Hindi‑speaking elders
[32]	Iran (Persian)	ABC‑16 (ABC‑F)	308	Factor structure, validation, reliability (Persian ABC)	Community‑dwelling older adults of Arak city	—	SV; IC; Rel; HT	α = 0.98; ICC = 0.85; split‑half = 0.95	Valid & reliable; acceptable temporal stability; one‑factor EFA
[33]	Iran (Persian)	ABC‑16 (older adults)	170	Determine validity & reliability of Persian ABC	Relatively healthy elders (no Parkinson’s/stroke/LL paralysis)	Model fit indices; test–retest	SV; IC; Rel	RMSEA = 0.08; CFI = 0.99; TLI = 0.99; α = 0.92; temporal rel.=0.83	Good fit & reliability in Persian elders
[34]	Israel (Arabic)	ABC‑16 (A‑ABC)	60	Arabic translation & psychometrics in ambulatory elders	≥65y; independent; no major conditions affecting balance; adequate vision/hearing	LLFDI; SF‑36; TUG; BBS	CV; SV; IC; Rel; ME; HT	α = 0.97; ICC = 0.98; low SEM/MDC	Excellent psychometrics; ceiling effects noted
[35]	Brazil (Brazilian‑Portuguese)	ABC‑16 & ABC‑6	158	Validate Brazilian ABC versions; determine cut‑offs; determinants	≥65y; stand unsupported; no cognitive impairment/exclude rehab hx; neuro/uncontrolled systemic dz	FES‑I; BBS; mCTSIB; US; 4‑m walk; GDS	SV; IC; Rel; HT; (ME partial)	Excellent α and ICC; cut‑offs ≤ 67% (ABC‑16) and ≤44% (ABC‑6)	Strong reliability; practical thresholds for impairment
[36]	China (Mandarin)	ABC‑6 vs ABC‑16	391	Psychometrics of Chinese ABC‑6; predictive value for falls	≥60y; ambulatory; exclusions for cognitive or major illness	BBS; Mini‑Cog; prospective falls	SV; IC; Rel; HT; ME	α = 0.938; ICC = 0.964; one‑factor EFA; cut‑off ≤60% (sens 71%, spec 84%)	Valid & reliable; predicts future falls
[37]	South Africa (Sepedi)	ABC‑16 (ABC‑S)	32	Translate to Sepedi; evaluate reliability	60–88y; rural; ambulatory; multistep instructions/exclude medical conditions ↑ fall risk	—	CV; Rel	ICC≈0.85 (test–retest); 0.81 (inter‑rater)	Reliable tool for Sepedi speakers; high mean ABC score (~81%)
[38]	Pakistan (Urdu)	ABC‑16 (Urdu)	120	Translate to Urdu; assess psychometric properties	≥60y; ambulatory; Urdu proficient/dizziness pathologies; psych disorders; vision impairments; fracture hx	FES‑I (Urdu)	CV; IC; Rel; ME; HT	α > 0.90; SEM = 2.40; MDC95 = 6.4; r(FES‑I)=−0.80	Reliable/valid; interpretable change values reported
[39]	Singapore (English)	ABC‑16	131	Convergent & predictive validity vs FES‑I and BRC	≥65y; independent; exclude cognitive impairment or assist-needed ambulation	FES‑I; BRC; regression models	HT (convergent/predictive)	r(ABC,FES‑I)=−0.794; r(ABC,BRC) = 0.642; ABC predicts FES‑I (R^2^ = 0.628)	Strong convergent validity; ABC outperforms BRC in predicting FES‑I

Note. Abbreviations—ABC = Activities‑specific Balance Confidence scale; ABC‑16 = 16‑item ABC; ABC‑6 = 6‑item short form; ABC‑S = simplified/IRT version; ABC‑NL = Dutch version; A‑ABC = Arabic version; ABC‑C = Cantonese version; ABC‑H = Hindi version; ABC‑*P* = Persian version (Hassan et al.); ABC‑F = Persian (Farsi) version (Khajavi et al.); CV = Content validity; SV = Structural validity; IC = Internal consistency; Rel = Reliability; ME = Measurement error; CCV/MI = Cross‑cultural validity/measurement invariance; HT = Hypotheses testing (construct validity); CRV = Criterion validity; RESP = Responsiveness; FES = Fall Efficacy Scale; FES‑I = Falls Efficacy Scale‑International; SF‑8 = 8‑item Short‑Form Health Survey; SF‑36 = 36‑item Short‑Form Health Survey; Katz ADL = Katz Index of Independence in Activities of Daily Living; MMSE = Mini‑Mental State Examination; PASE = Physical Activity Scale for the Elderly; PSE = Physical Self‑Efficacy scale; UST = Unipedal Stance Time; FR = Functional Reach; TUG = Timed Up and Go; MSL = Maximum Step Length; BBT = Berg Balance Test; BBS = Berg Balance Scale; GDS = Geriatric Depression Scale; ETGUG = Expanded Timed Get Up and Go; CFRSI = Comprehensive Falls Risk Screening Instrument; LLFDI = Late‑Life Function and Disability Instrument; mCTSIB = Modified Clinical Test of Sensory Interaction on Balance; US = Unilateral Stance; Mini‑Cog = Mini‑Cognitive Assessment; ROC = Receiver Operating Characteristic; AUC = Area Under the Curve; SEM = Standard Error of Measurement; SDC = Smallest Detectable Change; MDC95 = Minimal Detectable Change at 95% confidence; MIC = Minimal Important Change; ICC = Intraclass Correlation Coefficient; α = Cronbach’s alpha; *r* = Pearson correlation coefficient; R^2^ = Coefficient of determination; EFA = Exploratory Factor Analysis; CFA = Confirmatory Factor Analysis; CFI = Comparative Fit Index; TLI = Tucker–Lewis Index; RMSEA = Root Mean Square Error of Approximation; ADLs = Activities of Daily Living; QoL = Quality of Life; dz = disease(s); PT = physiotherapy; hx = history

### Risk of Bias

Overall methodological quality varied across studies (Table 2). Most were rated adequate to very good for internal consistency and reliability. Structural validity was supported in several versions but was indeterminate or doubtful in others due to limited sample size or incomplete reporting. Content validity was rarely addressed, and responsiveness was infrequently tested. According to the “worst score counts” principle, the most common limitations were inadequate reporting of content validity and lack of longitudinal designs for responsiveness.

**Table 2:** Results of risk of Bias

Study (Year)	Country/Language	Version	PD	CV	SV	IC	CCV/MI	REL	ME	HT	CRV	RESP	Overall RoB	Domain Bias	Key Notes
[18]	Korean/South Korea	ABC-16	D	D	A	VG	A	A	NA	A	NA	NA	D	B1,B2	Convenience sample; limited PROM development detail
[19]	Dutch/Netherlands	ABC-NL	I	I	A	VG	I	NA	NA	A	NA	NA	I	B1, B2, B5	No test–retest; limited content/PROM development
[20]	Chinese-Canadian/Canada	ABC-16	I	I	A	VG	D	VG	NA	A	NA	NA	I	B1, B2	Older translation methods; limited PROM development detail
[21]	Cantonese/Hong Kong	ABC-16	A	A	A	VG	D	VG	NA	A	NA	NA	D	B5	ME/MI/RESP not examined
[22]	English/Canada	ABC-S (simplified/IRT)	A	A	A	A	NA	A	NA	A	NA	NA	A	B1,B2,B3,B4, B6, B8	No test–retest; IRT development but limited reporting
[23]	English/USA	ABC-16	NA	D	A	VG	NA	VG	NA	A	NA	NA	D	B2,	Specific risk cohort; generalizability limits
[24]	English/USA	ABC-6	D	D	A	VG	NA	A	NA	A	NA	A	D	B1,B2	Small sample; limited content validity detail
[25]	Mandarin/China	ABC-16	I	I	I	D	I	VG	A	A	NA	NA	I	B1, B2, B3, B5	Content validity not described; PROM dev not COSMIN‑conform
[26]	Brazilian-Portuguese/Brazil	ABC-16	I	I	NA	NA	D	A	NA	NA	NA	NA	I	B1,B2	Early adaptation; limited validity testing
[27]	German/Germany	ABC-6	D	D	A	VG	D	VG	NA	A	NA	NA	D	B1,B2,B5	No measurement error; limited PROM dev info
[28]	English/USA	ABC-16	NA	NA	NA	VG	NA	D	NA	NA	NA	NA	D	B6,	Small sample; long retest interval
[29]	Persian/Iran	ABC-16	A	A	NA	VG	A	VG	NA	NA	NA	NA	A	B1,B2,B5,	ME/MI/RESP not examined
[30]	English/USA	ABC-6	NA	NA	NA	VG	NA	A	NA	A	NA	NA	A	B6,B8,	Limited reporting of ME/RESP
[31]	Hindi/India	ABC-H (Hindi)	A	A	NA	A	NA	VG	VG	A	NA	NA	A	B1, B2, B4, B8	No measurement error/resp
[32]	Persian/Iran	ABC-16	D	D	VG	VG	NA	VG	NA	NA	NA	NA	D	B1, B2	Limited content validity reporting
[33]	Persian/Iran	ABC-16	D	D	VG	VG	NA	VG	NA	NA	NA	NA	D	B1, B2	Limited broader validity testing
[34]	Arabic/Israel	ABC-16	A	A	A	VG	D	VG	VG	A	NA	NA	D	B5	One-site sample
[35]	Brazilian-Portuguese/Brazil	ABC-16 & ABC-6	D	D	A	VG	D	VG	D	A	NA	NA	D	B1,B2,B5,B7	ME/Ml limited
[36]	Mandarin/China	ABC-6	D	D	A	VG	A	VG	A	A	NA	NA	D	B1,B2,	Prospective component strengthens HT
[37]	Sepedi/South Africa	ABC-16	A	A	NA	NA	A	VG	NA	NA	NA	NA	A	B1,B2,B5	Small sample; minimal validity testing
[38]	Urdu/Pakistan	ABC-16	A	A	D	VG	A	VG	A	A	NA	NA	D	B1,B2, B5,B7,B8	Responsiveness not tested
[39]	English/Singapore	ABC-16	NA	NA	NA	NA	NA	NA	NA	A	NA	NA	A	B8	No reliability reported

Abbreviation: B = Boxes| PD = PROM development | CV = Content validity | SV = Structural validity | IC = Internal consistency | CCV/MI = Cross-cultural validity/Measurement invariance | REL = Reliability | ME = Measurement error | HT = Hypotheses testing | CRV = Criterion validity | RESP = Responsiveness | OVR = Overall (worst-score) | DB = Driver boxes | VG = Very good (Evidence standard met; preferred method optimally used) | A = Adequate (Standard assumably met; method not optimally applied) | D = Doubtful (Unclear if standard is met or preferred method used) | I = Inadequate (Evidence standard not met or method inadequate) | NA = Not applicable (Not relevant for the study/box)

### Measurement properties

Internal consistency was consistently sufficient, with Cronbach’s α ranging from 0.86 to 0.99 across languages and versions. Test–retest reliability was also strong, with ICC values between 0.82 and 0.99. Structural validity was generally supported: the Persian version demonstrated good CFA fit indices; the Cantonese versions confirmed one-dimensionality; and the Mandarin version indicated a possible two-factor structure. Measurement error was reported in only three languages: Arabic (SEM≈3.5%, MDC95≈9.6%), and Urdu (SEM≈2.4, MDC95≈6.4). Cross-cultural validity was assessed in some translations (Brazilian, Persian, German, Urdu, Sepedi), but formal invariance testing was rarely conducted. Construct validity was typically confirmed through moderate-to-strong correlations with related instruments such as the Falls Efficacy Scale, FES-I, SF-36, and measures of mobility. Responsiveness was seldom tested and remains a major evidence gap.

### Interpretability and feasibility

Several studies reported interpretability indices. Cutoff values were suggested in Brazilian Portuguese versions (ABC-16 ≤ 67%, ABC-6 ≤ 44%) and Chinese versions (ABC-6 ≤ 60% for predicting fallers). Ceiling effects were noted in the Arabic version. Minimal detectable change values were available in Arabic, and Urdu, providing benchmarks for clinical interpretation. In terms of feasibility, shorter versions (ABC-6, ABC-S) reduced administration time compared to the full ABC-16, with some evidence that the ABC-6 may be equally or more predictive of falls.

### Summary of findings

Based on COSMIN and GRADE synthesis (Table 3), the Arabic ABC-16 was categorized as Category A (ready for use). Versions in Chinese Canadian, Cantonese, and Mandarin ABC-6, Brazilian Portuguese ABC-16 and ABC-6 Portuguese [35], Persian, Urdu, German, Hindi, Sepedi, and English/Canadian demonstrated promising properties but require additional validation and were classified as Category B. The Dutch and Mandarin ABC-16 versions, and earlier Brazilian Portuguese [26], had weaker or inconsistent evidence and were classified as Category C (not recommended until re-validated). The Marques et al. [26] Brazilian Portuguese adaptation was also classified as Category C, as it examined intra/inter-rater reliability only and lacked structural, content, and construct validity evidence.

**Table 3:** Property assessment by study with recommendation (A/B/C)

Study/Year Year	Language/Country	Version	CV	SV	IC	CCV/MI	REL	ME	HT	CRV	RESP	Recommendation (A/B/C)	Overall Highlights
[18]	Korean/South Korea	ABC-16	?	+	+	+	?	?	+	?	?	B	Strong reliability, good construct validity
[19]	Dutch/Netherlands	ABC-NL	–	+	+	?	?	?	+	?	?	C	Good internal consistency; weak reliability evidence
[20]	Chinese-Canadian/Canada	ABC-16	–	+	+	+	?	?	+	?	?	B	Strong reliability; weak content validity
[21]	Cantonese/Hong Kong	ABC-16	+	+	+	+	?	?	+	?	?	B	Excellent α/ICC and structure, but ME/MI/responsiveness under-reported
[22]	English/Canada	ABC-S	+	+	+	+	?	?	+	?	?	B	IRT version promising; needs replication
[23]	English/USA	ABC-16	?	+	+	+	?	?	+	?	?	B	Good psychometrics
[24]	English/USA	ABC-6	?	+	+	+	?	?	+	?	+	B	ABC-6 efficient, predictive
[25]	Mandarin/China	ABC-16	–	–	?	+	?	–	+	?	?	C	Very strong reliability; weak validity
[26]	Brazilian-Portuguese/Brazil	ABC-16	?	+	+	?	+	?	?	NA	?	C	Early adaptation; limited validity reporting (later studies strengthen)
[27]	German/Germany	ABC-6	?	+	+	+	?	?	+	?	?	B	Excellent reliability/validity; short efficient form
[28]	English/USA	ABC-16	?	?	+	+	?	?	+	?	?	B	Excellent reliability; redundancy possible
[29]	Persian/Iran	ABC-16	–	?	?	+	?	?	?	?	?	B	Good α/ICC; limited ME/MI
[30]	English/USA	ABC-6	+	?	+	?	+	+	+	NA	?	B	Useful for underserved older adults
[31]	(Hindi)/ India	ABC‑H	?	+	+	?	+	?	+	NA	?	B	Rigorous translation; α ≈ 0.99 and ICC≈0.97 with SEM/MDC reported; strong convergent validity; needs SV/MI and responsiveness.
[32]	Persian/Iran	ABC‑16	+	+	+	+	+	+	+	?	?	B	Very high α and acceptable ICC; one-factor EFA; content validity and ME/RESP minimally reported.
[33]	Persian/ Iran	ABC‑16	?	+	+	+	?	?	+	?	?	B	CFA confirms one-factor structure; α = 0.92 and ICC = 0.83; good construct validity; content validity and ME/RESP reporting limited.
[34]	Arabic/Israel	ABC-16	?	+	+	+	+	?	+	?	?	A	One of the strongest adaptations; excellent psychometric support
[35]	Brazilian-Portuguese/Brazil	ABC-16 & ABC-6	+	?	?	+	?	+	?	?	?	B	Strong reliability; practical cut-offs; ME/MI patchy
[36]	Mandarin/China	ABC-6	+	?	+	+	+	+	+	?	?	B	Strong predictive validity and reliability; clear structure
[37]	Sepedi/South Africa	ABC-16	?	?	?	?	?	?	+	?	?	B	Promising; needs structural/construct follow-through
[38]	Urdu/Pakistan	ABC-16	+	?	+	+	+	+	+	?	?	B	Reliable/valid; add MI and longitudinal responsiveness
[39]	English/Singapore	ABC-16	?	?	?	?	?	?	+	?	?	B	Strong convergent & predictive validity in Singapore cohort

Note. Recommendation (A/B/C): A: Sufficient content validity and internal consistency; other properties mostly acceptable, B: Promising; strengthen MI/ME/responsiveness locally, C: Content validity insufficient or major methodological concerns

Overall, reliability and internal consistency were consistently strong across all versions. Structural validity was generally adequate, but content validity and responsiveness remain underreported. Emerging evidence supports interpretability indices and predictive validity, particularly for the ABC-6, which shows potential as a brief screening tool for fall risk in older.

### ABC cross-version comparisons

Across all versions combined: Internal consistency, reliability, structural validity, and hypotheses testing remain sufficient (+) with moderate certainty. Content validity stays inconsistent (±) with low certainty, mainly because many translations did not follow full COSMIN development procedures (limited patient/clinician qualitative work). Measurement error is sufficient (+) but still supported by few studies, so certainty remains moderate. Criterion validity and responsiveness are largely indeterminate (?) due to the lack of a gold standard and scarce longitudinal testing. (Table 4). ABC-16 (full scale): Moderate-certainty evidence for internal consistency, reliability, structural validity, hypothesis testing, and cross-cultural validity/measurement invariance. Very low certainty for criterion validity and responsiveness. (Table 5) ABC-6 (short form): Moderate-certainty evidence for internal consistency, reliability, structural validity, and hypothesis testing. Very low for cross-cultural validity and responsiveness. (Table 6).ABC-S (simplified/IRT): Evidence remains limited (single-study), suggesting sufficient structure/reliability/validity but low to very low overall certainty (Table 7).

**Table 4:** Overall measurement properties across all ABC versions (*n* = 22)

Measurement Property	k (judgeable)	+	–	?	Summarized Rating	GRADE (certainty)
CV	22	7	4	11	±	Low
SV	22	13	1	8	+	Moderate
IC	22	18	0	4	+	Moderate
Rel	22	20	0	2	+	Moderate
ME	22	4	0	18	+	Moderate
CCV/MI	22	4	1	17	+	Moderate
HT	22	17	0	5	+	Moderate
CRV	22	0	0	22	?	Very low
Resp	22	1	0	21	?	Very low

Note. COSMIN summarized ratings use + (sufficient), – (insufficient), ± (inconsistent), and? (indeterminate)

GRADE reflects certainty of evidence. CV = Content validity | SV = Structural validity |

IC = Internal consistency | CCV/MI = Cross-cultural validity/Measurement invariance |

REL = Reliability | ME = Measurement error | HT = Hypotheses testing | CRV = Criterion validity | RESP = Responsiveness

**Table 5:** ABC-16 (full): summarized results, summarized ratings, and GRADE (*n* = 16)

Measurement Property	k (judgeable)	+	–	?	Summarized Rating	GRADE (certainty)
CV	16	6	4	6	±	Low
SV	16	8	1	7	+	Moderate
IC	16	12	0	4	+	Moderate
Rel	16	14	0	2	+	Moderate
ME	16	3	0	13	+	Moderate
CCV/MI	16	4	1	11	+	Moderate
HT	16	11	0	5	+	Moderate
CRV	16	0	0	16	?	Very low
Resp	16	0	0	16	?	Very low

Note. COSMIN summarized ratings use + (sufficient), – (insufficient), ± (inconsistent), and? (indeterminate)

GRADE reflects certainty of evidence. CV = Content validity | SV = Structural validity |

IC = Internal consistency | CCV/MI = Cross-cultural validity/Measurement invariance |

REL = Reliability | ME = Measurement error | HT = Hypotheses testing | CRV = Criterion validity | RESP = Responsiveness

**Table 6:** ABC-6 (short): summarized results, summarized ratings, and GRADE

Measurement Property	k (judgeable)	+	–	?	Summarized Rating	GRADE (certainty)
CV	5	0	0	5	?	Very low
SV	5	4	0	1	+	Moderate
IC	5	5	0	0	+	Moderate
Rel	5	5	0	0	+	Moderate
ME	5	1	0	4	+	Low
CCV/MI	5	0	0	5	?	Very low
HT	5	5	0	0	+	Moderate
CRV	5	0	0	5	?	Very low
Resp	5	1	0	4	?	Low

Note. COSMIN summarized ratings use + (sufficient), – (insufficient), ± (inconsistent), and? (indeterminate)

GRADE reflects certainty of evidence. CV = Content validity | SV = Structural validity |

IC = Internal consistency | CCV/MI = Cross-cultural validity/Measurement invariance | REL = Reliability |

ME = Measurement error | HT = Hypotheses testing | CRV = Criterion validity | RESP = Responsiveness

**Table 7:** ABC-S (simplified): summarized results, summarized ratings, and GRADE

Measurement property	k (judgeable)	+	–	?	Summarized rating	GRADE (certainty)
CV	1	1	0	0	+	Low
SV	1	1	0	0	+	Low
IC	1	1	0	0	+	Low
Rel	1	1	0	0	+	Low
ME	1	0	0	1	?	Very low
CCV/MI	1	0	0	1	?	Very low
HT	1	1	0	0	+	Low
CRV	1	0	0	1	?	Very low
Resp	1	0	0	1	?	Very low

Note. COSMIN summarized ratings use + (sufficient), – (insufficient), ± (inconsistent), and? (indeterminate)

GRADE reflects certainty of evidence. CV = Content validity | SV = Structural validity |

IC = Internal consistency | CCV/MI = Cross-cultural validity/Measurement invariance | REL = Reliability |

ME = Measurement error | HT = Hypotheses testing | CRV = Criterion validity | RESP = Responsiveness

### Practice implications

The findings of this review suggest that the ABC scale can be used in both clinical and research settings, but the choice of version should be guided by the available evidence for each language and context.

**Category A (ready for use):** The Arabic ABC-16 demonstrated the strongest overall evidence across reliability, structural validity, and construct validity, with sufficient content validity and low risk of bias. These versions can be considered for use in practice with minimal additional validation, particularly in their respective cultural and linguistic contexts. Clinicians and researchers can apply these versions in clinical practice and outcome evaluation.

**Category B (use with caution):** Versions translated into Chinese-Canadian, Cantonese, and Mandarin ABC-6 version, Brazilian Portuguese ABC-16 and ABC-6 [35], Persian, German, Urdu, Sepedi, English/Canadian, and Hindi (ABC-H) generally showed excellent internal consistency and reliability. However, gaps remain in the evaluation of measurement error, cross-cultural validity, and responsiveness. These versions are suitable for use when no Category A instrument is available, but additional local testing is advised. For example, before widespread adoption, researchers should conduct small-scale content validity assessments (e.g., with older adults and clinicians) to confirm relevance, comprehensibility, and comprehensiveness of items. Moreover, documenting minimally important change (MIC) would strengthen their interpretability for clinical decision-making.

**Category C (re-validation required):** The Dutch and Mandarin versions of ABC-16 and the Brazilian Portuguese ABC-16 [26], which were limited to intra/inter-rater reliability only, did not meet current COSMIN expectations for PROM development or content validity. If these instruments need to be used in their current form, re-validation or redevelopment is strongly recommended, including modern translation procedures, cultural adaptation, and testing of measurement invariance.

## Discussion

To the best of our knowledge, this is the first COSMIN-based systematic review dedicated solely to the ABC scale across all versions and languages. By focusing on a single instrument, rather than comparing multiple PROMs, we were able to examine each version in depth and provide version-specific recommendations (A/B/C categories). In addition, we synthesized evidence on interpretability metrics such as cut-offs, SEM, and MDC, which enhance the clinical applicability of the ABC in both research and practice.

Across 22 studies comprising 3,397 participants conducted in 11 languages, the ABC demonstrated uniformly strong reliability and internal consistency, with Cronbach’s α values consistently above 0.86 and ICC values above 0.82. Structural validity was generally supported, with most studies confirming the unidimensional nature of the scale. Construct validity was typically adequate, with moderate-to-strong correlations between ABC scores and established measures such as the FES/FES-I, Berg Balance Scale, and mobility tests. These findings support the use of the ABC in clinical and research settings as a measure of balance confidence in community-dwelling older adults.

Despite these strengths, several evidence gaps were identified. Content validity procedures were inconsistently reported and often lacked systematic involvement of older adults and clinicians in the development or translation process, reducing certainty in this domain. Responsiveness, the ability of the ABC to detect meaningful change over time, was seldom tested, limiting confidence in its use as an outcome measure for interventions. Similarly, cross-cultural measurement invariance was rarely assessed, making it unclear whether ABC scores can be validly compared across languages or cultural groups. Measurement error was addressed in only a small subset of translations (Arabic, Urdu), leaving gaps in interpretability for many versions.

It is useful to situate the ABC within the broader PROM landscape. The Falls Efficacy Scale–International (FES-I) remains the guideline-recommended PROM for concerns about falling, emphasizing the emotional and behavioral aspects of worry and avoidance [40]. In contrast, the ABC reflects balance confidence and fall-related self-efficacy, constructs that are particularly relevant for active, community-dwelling older adults. Studies focused on neurological populations have demonstrated the predictive value of the ABC, with scores emerging as stronger independent predictors of future falls than fall history, underlying pathology, or performance-based balance tests [41]. The recently developed Balance Recovery Confidence (BRC) Scale captures a distinct domain, balance recovery confidence, or the perceived ability to arrest a fall after a slip, trip, or perturbation [42]. Importantly, these instruments should not be used interchangeably, as each maps onto a different falls-related psychological construct [8, 39]. While FES-I best captures concern, ABC remains the most extensively validated tool for confidence, and BRC offers a novel means of targeting recovery-specific self-efficacy. These distinctions highlight the importance of selecting PROMs according to the construct of interest rather than defaulting to convenience.

Our findings align with and extend those of Soh et al. [13], who reported sufficient structural validity for several ABC variants (ABC-6, ABC-15/ABC-S, and ABC-16) with moderate-to-high quality evidence. Consistent with their review, we also found that content validity was under-studied and typically of low or very low quality. Soh et al. [13] further highlighted practical constraints, such as language limitations, that hindered full appraisal of certain versions (e.g., German, Dutch, Persian) and emphasized the need for qualitative work with older adults and clinicians to strengthen content validity [13]. We extend their review by including studies published up to July 2025, applying the full COSMIN methodology, and providing actionable A/B/C recommendations. Compared with earlier reviews. [12, 43]. Our review offers standardized COSMIN/GRADE evaluation, cross-cultural mapping, and consolidated clinical thresholds.

The COSMIN framework with its GRADE-adapted approach proved highly suitable, offering a transparent, standardized way to assess PROMs and grade certainty of evidence. The most common risk-of-bias drivers were weak content validity, small sample sizes for factor analyses, limited reporting of test–retest intervals, and lack of predefined hypotheses for construct validity. For clinicians, the identification of Category A versions (Arabic ABC-16) provides immediate guidance for implementation. Cut-offs (e.g., ≤67% for ABC-16, ≤44–60% for ABC-6) [35, 36] and available MDC values (Arabic, Urdu) further enhance interpretability in practice. However, ceiling effects have been observed in fitter populations [34, 44], suggesting that very high scores should be interpreted with caution and ideally paired with a performance-based test. Taken together, these findings mean the ABC can be applied in practice, as a feasible screening and monitoring tool in community-dwelling adults and clinical programs, provided it is used alongside complementary assessments and interpreted within the local cultural and functional context. It is important to emphasize that the ABC scale should not be used in isolation, but rather integrated into a multifactorial falls risk assessment and prevention program that considers psychological, physical, and environmental factors together. Future research should strengthen content validity through qualitative work, undertake formal cross-cultural invariance testing, and conduct longitudinal studies to establish responsiveness and minimally important change.

### Study limitations

This review has several limitations that should be acknowledged. First, many included studies had small to moderate sample sizes, limiting the precision of estimates for structural validity, reliability, and cross-cultural equivalence. Second, methodological quality varied substantially; in line with COSMIN guidance, most studies were rated as inadequate or doubtful for content validity, which is considered the most important measurement property. Because content validity underpins all other properties, this gap limits the strength of conclusions across languages.

Third, evidence for responsiveness and cross-cultural validity was scarce, and only a minority of studies reported measurement error indices such as SEM or MDC. Fourth, heterogeneity in study designs, populations, and statistical approaches prevented quantitative pooling of results, so synthesis was narrative rather than meta-analytic. Finally, although a comprehensive search strategy was used, this review was limited to peer-reviewed publications, and grey literature or unpublished validations may have been missed. We also excluded studies conducted in populations with specific neurological conditions (e.g., Parkinson’s disease, stroke, multiple sclerosis), which narrows generalisability but ensures focus on community-dwelling older adults. This review focused on community-dwelling older adults, rather than condition-specific cohorts, to evaluate the ABC as a general screening tool rather than a disease-specific instrument. While this approach improves internal consistency, it may limit generalizability to populations with complex chronic conditions.

Despite these limitations, this review provides the most comprehensive synthesis to date of the ABC scale in community-dwelling older adults and highlights clear priorities for future research, including rigorous evaluations of content validity, responsiveness, and cross-cultural invariance.

## Conclusion

This COSMIN-based systematic review demonstrates that the ABC scale is a reliable and valid self-report measure of balance confidence among community-dwelling older adults. Among the available adaptations, the Arabic ABC-16 can be used in clinical and research settings. Other versions, including those in Brazilian Portuguese, Persian, Urdu, German, Cantonese, Hindi, Sepedi, and English/Canadian, demonstrated promising properties but require further validation. Dutch and Mandarin versions should not be used until re-validated. Overall, the ABC scale represents a valuable patient-reported outcome measure for identifying and monitoring balance confidence in older adults worldwide. With stronger attention to content validity and responsiveness, the ABC has the potential to become a useful tool with strong evidence for fall risk assessment and prevention programs.

## Data Availability

No datasets were generated or analysed during the current study.

## Abbreviations

ABC: Activities-specific Balance Confidence scale
ABC-16: 16-item version of the ABC scale
ABC-6: 6-item short version of the ABC scale
ABC-S: Simplified/IRT version of the ABC scale
ABC-NL: Dutch version of the ABC scale
A-ABC: Arabic version of the ABC scale
ABC-C: Cantonese version of the ABC scale
ABC-H: Hindi version of the ABC scale
ABC-P: Persian version of the ABC scale
ABC-F: Persian (Farsi) version of the ABC scale
ADL: Activities of Daily Living
AUC: Area Under the Curve
BBS: Berg Balance Scale
BBT: Berg Balance Test
BRC: Balance Recovery Confidence scale
CFA: Confirmatory Factor Analysis
CFI: Comparative Fit Index
CCV/MI: Cross-cultural validity/Measurement invariance
COSMIN: COnsensus-based Standards for the selection of Health Measurement Instruments
CRV: Criterion validity
CV: Content validity
DB: Driver boxes
dz: Disease(s)
EFA: Exploratory Factor Analysis
ETGUG: Expanded Timed Get Up and Go
FES: Falls Efficacy Scale
FES-I: Falls Efficacy Scale–International
FR: Functional Reach
GDS: Geriatric Depression Scale
GRADE: Grading of Recommendations Assessment Development and Evaluation
HT: Hypotheses testing (construct validity)
IC: Internal consistency
ICC: Intraclass Correlation Coefficient
IRT: Item Response Theory
LLFDI: Late-Life Function and Disability Instrument
MDC95: Minimal Detectable Change at 95% confidence
ME: Measurement error
MIC: Minimal Important Change
MMSE: Mini-Mental State Examination
mCTSIB: Modified Clinical Test of Sensory Interaction on Balance
NA: Not applicable
PASE: Physical Activity Scale for the Elderly
PROM: Patient-Reported Outcome Measure
PT: Physiotherapy
QoL: Quality of Life
Rel: Reliability
RESP: Responsiveness
RMSEA: Root Mean Square Error of Approximation
ROC: Receiver Operating Characteristic
SDC: Smallest Detectable Change
SEM: Standard Error of Measurement
SF-8: 8-item Short Form Health Survey
SF-36: 36-item Short Form Health Survey
SV: Structural validity
TLI: Tucker–Lewis Index
TUG: Timed Up and Go test
UST: Unipedal Stance Time
